# Adenovirus Armed With TNFa and IL2 Added to aPD-1 Regimen Mediates Antitumor Efficacy in Tumors Refractory to aPD-1

**DOI:** 10.3389/fimmu.2021.706517

**Published:** 2021-07-23

**Authors:** Victor Cervera-Carrascon, Dafne C. A. Quixabeira, Joao M. Santos, Riikka Havunen, Ioanna Milenova, Jan Verhoeff, Camilla Heiniö, Sadia Zafar, Juan J. Garcia-Vallejo, Victor W. van Beusechem, Tanja D. de Gruijl, Aino Kalervo, Suvi Sorsa, Anna Kanerva, Akseli Hemminki

**Affiliations:** ^1^ Cancer Gene Therapy Group, Translational Immunology Research Program, Faculty of Medicine, University of Helsinki, Helsinki, Finland; ^2^ TILT Biotherapeutics Ltd, Helsinki, Finland; ^3^ Department of Medical Oncology, Amsterdam University Medical Centers, Vrije Universiteit Amsterdam, Cancer Center Amsterdam, Amsterdam Infection & Immunity Institute, Amsterdam, Netherlands; ^4^ Orca Therapeutics, Amsterdam, Netherlands; ^5^ Department of Molecular Cell Biology & Immunology, Amsterdam Infection & Immunity Institute and Cancer Center Amsterdam, Amsterdam UMC, Amsterdam, Netherlands; ^6^ Department of Obstetrics and Gynecology, Helsinki University Hospital, Helsinki, Finland; ^7^ Helsinki University Hospital Comprehensive Cancer Center, Helsinki, Finland

**Keywords:** cancer immunotherapy, oncolytic virus, adenovirus, checkpoint inhibitor resistance, tumor microenvironment

## Abstract

Immune checkpoint inhibitors such as anti-PD-1 have revolutionized the field of oncology over the past decade. Nevertheless, the majority of patients do not benefit from them. Virotherapy is a flexible tool that can be used to stimulate and/or recruit different immune populations. T-cell enabling virotherapy could enhance the efficacy of immune checkpoint inhibitors, even in tumors resistant to these inhibitors. The T-cell potentiating virotherapy used here consisted of adenoviruses engineered to express tumor necrosis factor alpha and interleukin-2 in the tumor microenvironment. To study virus efficacy in checkpoint-inhibitor resistant tumors, we developed an anti-PD-1 resistant melanoma model *in vivo*. In resistant tumors, adding virotherapy to an anti-PD-1 regimen resulted in increased survival (p=0.0009), when compared to anti-PD-1 monotherapy. Some of the animals receiving virotherapy displayed complete responses, which did not occur in the immune checkpoint-inhibitor monotherapy group. When adenoviruses were delivered into resistant tumors, there were signs of increased CD8 T-cell infiltration and activation, which - together with a reduced presence of M2 macrophages and myeloid-derived suppressor cells - could explain those results. T-cell enabling virotherapy appeared as a valuable tool to counter resistance to immune checkpoint inhibitors. The clinical translation of this approach could increase the number of cancer patients benefiting from immunotherapies.

## Introduction

The discovery of immune checkpoint pathways such as the CTLA-4 and PD-1 axes, and their subsequent blockade by inhibiting antibodies (including, but not restricted to, anti-CTLA-4, anti-PD-1 and anti-PD-L1) has revolutionized the field of immunotherapy for cancer (1, 2). However, only a limited number of patients benefit from these therapies. In the majority of tumor types response rates in unselected populations are usually between 10%-40% (3, 4). Even in responding patients, resistance often develops, leading to disease progression. Thus, there is an obvious room for improvement (5, 6). Some of those patients seem to be intrinsically refractory to the immune checkpoint inhibitors (ICIs) while others stop responding after an initial response (7). Therefore, ICI refractory patients present a tremendous unmet clinical need.

Studies trying to unveil the cause of resistance to ICIs point to multiple possibilities including the presence of mutations in tumors that render them insusceptible to cytotoxic immune mechanisms (*e.g.* mutations in PTEN, EGFR, MYC, JAK1/2, β2M, *etc.*) *(* 8–10). Others suggest that resistance mechanisms do not necessarily stem from genetic mutations in the tumor but may derive from immunosuppressive conditions that hamper development of antitumor immunity (*e.g.* T-cell exhaustion, altered metabolism, expression of alternative inhibitory receptors, *etc.*) *(* 11–13). The most likely scenario is that both explanations can be true for different tumors.

In a different corner of immunotherapy of cancer are oncolytic viruses; a tool partially discovered over a hundred years ago. However, their immunological effects were initially ignored, resulting in only moderate efficacy results (14). Enthusiasm for oncolytic viruses was revived with the blooming of immunotherapy for cancer, as they were found to trigger broad immune responses that can counter immune suppressive conditions in the tumor microenvironment (15, 16). Simultaneously, they deliver direct lytic effects on tumor cells, which helps to reduce tumor burden and induce antigen release in an immunogenic fashion. However, what makes them such a versatile tool are the near-infinite possibilities granted through genetic modification (14, 17, 18). This way, each virus can be armed to overcome specific efficacy limitations and hurdles in the tumor microenvironment or to activate different immune cell types.

An oncolytic adenovirus Ad5/3-E2F-d24-hTNFa-IRES-hIL2 (also known as TILT-123) was engineered for enhanced entry into tumor cells (19, 20), highly selective tumor replication (21) and to enable anti-tumor T-cell responses (22–24). The two cytokines (TNFa and IL-2) were chosen empirically in a data driven manner as the optimal arming devices in the context of T cells (22–24). Besides the arming devices encoded by the virus, the highly immunogenic nature of adenoviruses makes them a particularly good candidate to engage the T-cell compartment in tumors (25, 26). This makes them a strong candidate for boosting T-cell activities such as those needed for the activity of ICIs.

Clinical studies have shown a positive impact on response rates against solid tumors when an oncolytic virus was used together with checkpoint blockade (27, 28). Moreover, we have shown pre-clinically that adenoviruses engineered to express TNFa and IL-2 synergize with checkpoint inhibitors (29) with promising results. Synergy was mediated by increased trafficking of anti-tumor immune cells into the tumor niche after virotherapy. Conversely, ICIs protected effector T cells from reactive suppressive signals.

In essence, each counterpart of this approach complements the weakness of the other. Anti-PD-1 therapy requires the presence of T cells to work, which can achieved by the armed virus. Any strong immune reaction against the tumor, including the one mediated by the virus, causes a compensatory immune suppressive counter-reaction, neutralized by the anti-PD-1 in this setting.

To study if the use of virotherapy could be a valid approach in ICI-resistant patients, which currently present tremendous unmet clinical need, we developed an *in vivo* model refractory to anti-PD-1. We then applied treatment with cytokine-armed adenoviruses, with or without anti-PD-1. The aim of the study was to evaluate whether the use of virotherapy could yield therapeutic benefit in ICI resistant conditions.

## Materials and Methods

### *In Vivo* Experiments

*In vivo* experiments were carried out in C57BL/6OlaHsd female mice, 4-6 week old by the initiation of the tests (purchased from Envigo Labs, Huntingdon, UK). 2.5 x 10^5^ B16.OVA melanoma cells were implanted subcutaneously in the left lower flank. When the minimum tumor size criterion of 4 mm was met, animals were randomized into different treatment groups. Tumor volumes were measured and overall health was assessed daily. Animals having open wounds (i.e. ulcers at the injection site) were euthanized. Maximum allowed tumor diameter was 18 mm, after which animals were immediately euthanized. Animals with no observable tumors were kept alive at least 90 days after they received the first treatment to ensure no tumor recurrence.

### Antibodies and Viruses

Treatment diagrams are provided for each specific experiment. APD-1 treatments consisted of systemically (intraperitoneally) delivered antibody dosed as 0.1 mg (clone 10F.9G2, BE0101, BioXCell, Lebanon, New Hampshire, USA) diluted in PBS. Virotherapy treatments consisted of 1 x 10^8^ viral particles (including equal amounts of Ad5-CMV-mIL2 and Ad5-CMV-mTNFa viruses, non-replicative in mice but historically used as model for the Ad5 man-based therapies which are replication competent in that host). The expression of TNFa and IL-2 after the use of the viruses in B16.OVA model has been studied before (23). In those experiments including intratumoral virus treatments, control groups not receiving viruses were injected intratumorally with an equivalent amount of PBS.

### Transcriptome Analyses

Tumors harvested from *in vivo* experiments were stabilized in RNAlater (R0901, Sigma-Aldrich, St. Louis, Missouri, USA) and stored at -20°C. Following RNeasy (74104, Qiagen, Hilden, Germany) kit manufacturer’s guide, RNA was purified from the tumor samples and the concentration was adjusted based on the amount of RNA detected with a spectrophotometer (Biophotometer, Eppendorf, Wesbury, New York, USA). Sequencing of the RNA samples was outsourced to BGI Tech Solutions (Tai Po, Hong Kong) that also performed data cleaning and quantitative analyses in a single blind manner.

### CyTOF

Tumors harvested from *in vivo* experiments were processed into single cell suspensions and stored in freezing media (including 10% dimethyl sulfoxide) until they were stained for mass cytometry analyses. The samples were thawed, and stained with 5uM cisplatin as a viability marker. The cells were fixed with 1.6% PFA (v/v) in PBS. Samples were barcoded with 20 unique palladium isotopes as per manufacturer’s protocol (Fluidigm). Samples were treated with Human TruStain FcX (Biolegend), and antibodies for cell surface staining were added first, followed by intracellular staining for nuclear and phospho-proteins. DNA was stained with 1:4000 iridium and fix-perm buffer (Fluidim) and stored overnight. Samples were acquired the following day on CyTOF3-Helios mass cytometer in double-distilled H2O spiked with 10% EQ four element Calibration Beads (Fluidigm) at a rate of 250-300 events per second. After acquisition sample files were normalized over time with the use of the calibration beads and deconvoluted into individual sample files through Fluidigm debarcoding software. A complete list of the metal-bound antibodies can be found as **Supplementary Table 1**.

### Statistical Analyses

GraphPad Prism 8 (GraphPad Software, San Diego, California, USA) analysis tools were used to perform the log rank Mantel-Cox test on Kaplan-Meier survival curves and Mann-Whitney test, as well as a means to generate graphical representations of the data. SPSS Statistics 25 (IBM, Armonk, New York, USA) was the software used for analyses on tumor growth evolution based on daily measures of the tumor diameters with a mixed model test as described before (29). Statistical significance was set for p-values under 0.05.

## Results

### B16.OVA Tumor Model Responds to Anti-PD-1 but Long-Term Efficacy Is Rare

**Figure 1:**
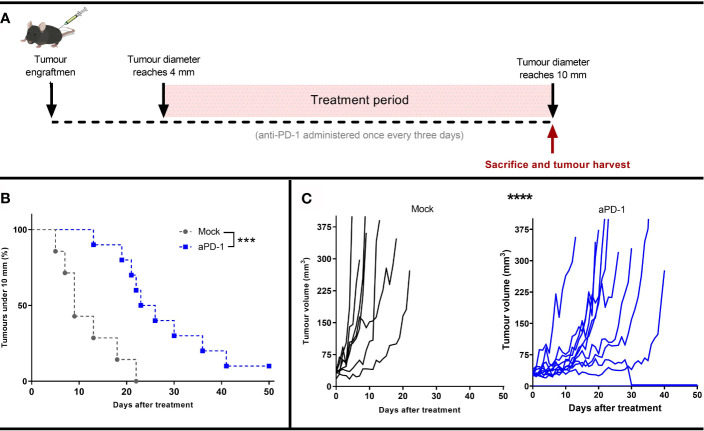
Development of an in *vivo* model refractory to aPD-1. **(A)** Experimental design: 17 mice were engrafted with subcutaneous B16.OVA tumors (2.5 x 10^5^ cells/animal). When those tumors reached 4 mm in maximum diameter, the animals were assigned to Mock (n=7) or to aPD-1 group (n=10). 0.1 mg of aPD-1 (or PBS) was given intraperitoneally every three days. When tumors progressed over 10 mm, animals were sacrificed. **(B)** Percentage of animals with a tumor under 10 mm after they started treatment. **(C)** Individual tumor growth curves for both groups. (Kaplan-Meier, Log rank Mantel-Cox test; ***p < 0.001, ****p < 0.0001).

To study the mechanism of resistance to aPD-1, B16.OVA, a model that displays limited response to PD-1 blocking antibodies (29, 30), was selected. Animals whose tumor reached at least 4 mm maximum diameter were randomly assigned to either “Mock” or “aPD-1” groups. Animals in the “aPD-1” group received PD-1 inhibiting antibody treatment systemically once every three days for at least 5 rounds. All the animals’ tumors were measured daily until the maximum diameter was at least 10 mm. Animals with tumors surpassing that threshold were euthanized and tumors collected for further analysis (**Figure 1A**).

While the size of tumors at the start of the treatment period and at the time of euthanasia were the same for both groups (**Supplementary Figure 1**), the time for the them to reach the 10 mm diameter threshold was significantly different in the two groups (p=0.0008) (**Figure 1B**). Whereas tumors from the “Mock” group took 5-22 days to reach the maximum allowed volume, for the “aPD-1” group this was 13-41 days, with the exception of one complete responder. Thus, aPD-1 treatment showed significant (p=0.0002) benefit in tumor growth control, and one out of the ten treated animals displayed an apparent complete response by day 30. Even though the treatment slowed tumor progression, 90% of the tumors eventually relapsed and reached the diameter threshold of 10 mm (**Figure 1C**).

These results show initial tumor growth control upon PD-1 blockade, but also the lack of long-term responses in most patients, similarly to what is seen with human patients with single agent ICI.

### Tumors That Grow Following Anti-PD-1 Blockade Show Different Gene Expression Profiles Than Growing Tumors Naïve to the Therapy

**Figure 2:**
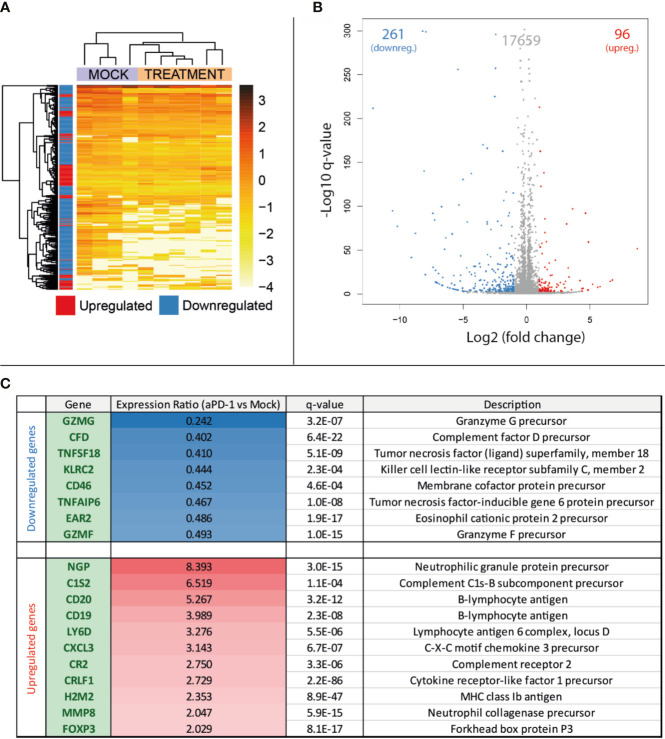
Comparison at the gene expression level of treatment naïve progressing tumors and tumors progressing after aPD-1 therapy. Animals treated as described in **Figure 1A** were sacrificed and tumors harvested when they were considered refractory to aPD-1. RNA was extracted and expression profiles from both groups were compared. **(A)** Heatmap and unsupervised clustering of samples. **(B)** Volcano plot for the expression level comparison between treatment naïve and aPD-1 treated tumors. **(C)** Immune related significantly regulated genes. (Differences in gene regulations were taken into account if fold change was ≤-2 or ≥2, with a q-value ≤ 0.001).

After collection of samples as described in **Figure 1A**, RNA was extracted for total RNA sequencing and gene expression quantification. For this analysis, four samples belonging to the “Mock” group and six samples from “aPD-1” group were randomly selected. After data cleaning, samples were arranged in a heatmap (**Figure 2A**) and clustered based on similarities between samples. This approach grouped samples from both groups apart with reasonable accuracy. The comparison of the expression profiles between groups identified 357 upregulated or downregulated genes in tumors treated with aPD-1 (**Figure 2B**). Out of those genes (**Supplementary Table 2**), 19 had a marked connection to the immune system, based on UniProt annotations (**Figure 2C**).

From the downregulated immune-related genes with established function, 75% were linked to T cells. Those genes included T-cell protease precursors (GZMG and GMZF), T-cell activity regulators (TNFSF18 [a.k.a. GITRL] and EAR2) and other T-cell proteins relevant in the interaction with other cell types (KLRC2) and immune components such as the complement (CD46). Some of the downregulated genes can also be related to other lymphocyte populations, such as NK cells or B cells.

Regarding the upregulated genes, most of them were related to the B-cell compartment (CD19, CD20, CR2, MMP8 and LY6D), followed by neutrophils (NGP, MMP8 and CXCL3). Complement-related genes were also noticeable in the upregulated genes (C1S2 and CR2). In contrast with the high number of T-cell related genes downregulated in aPD-1 refractory tumors, the only upregulated gene clearly associated with this cell population was FOXP3, a transcription factor characteristic of the regulatory T cell population.

Among the whole list of differentially expressed genes in the aPD-1 refractory tumors, there was an observable trend indicating suppression of T-cell related genes. Other cell populations were also affected but the results were not as clear.

### T-Cell Enabling Virotherapy Restores Anti-PD-1 Responses in Refractory Tumors

**Figure 3:**
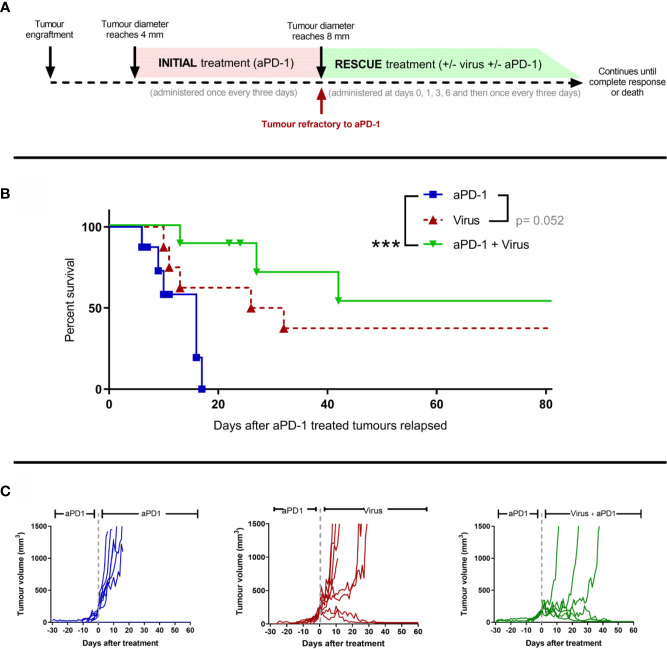
The use of an engineered adenovirus is able to trigger tumor growth control in aPD-1 refractory tumors. **(A)** Experimental design: 29 mice were engrafted subcutaneously with 2.5 x 10^5^ B16.OVA tumor cells. When those tumors reached 4 mm in maximum diameter, they started receiving 0.1 mg of aPD-1 every three days intraperitoneally. When tumors progressed over 8 mm, animals were assigned to a group where they were treated with the same aPD-1 regimen (n=8), with 1x108 VP intratumorally once every three days (n=8), or both (n= 8). Treatments continued until complete responses were observed or sacrifice criteria was reached. **(B)** Cancer-specific survival. **(C)** Individual tumor growth curves for the groups. (Kaplan-Meier, Log rank Mantel-Cox test; ***p < 0.001).

Next, we studied whether adenoviruses coding for two cytokines enhancing anti-tumor T-cell activity (TNFa and IL-2) could induce responses in the aPD-1 refractory tumors. Similarly as described in **Figure 1A**, animals carrying subcutaneous tumors were treated with aPD-1 (“initial treatment”) until their tumors progressed and fit the refractory criteria (**Figure 3A**). After the refractory status was achieved, animals were randomized into “aPD-1” group, where animals kept on receiving aPD-1, “Virus” group, where animals received only virotherapy, or “aPD-1 + Virus” group, where animals received aPD-1 and additionally virotherapy (“rescue treatment”). Treatments continued until the maximum ethically allowed tumor diameter (18 mm) or apparent complete responses (no visually noticeable lesions in the original tumor area).

With regard to tumor-specific survival after the tumors reached the refractory threshold (**Figure 3B**), When the anti-PD-1 regimen was combined with T-cell enabling virotherapy, survival significantly increased (p=0.0009) and even triggered complete responses in 50% of the animals. Virotherapy alone did not improve the survival significantly compared to aPD-1 as monotherapy, but it triggered complete responses in a fraction of the group. Similar conclusions can be drawn from the analysis of individual tumor growth curves (**Figure 3C**). Tumors in the “aPD-1” group took 6-17 days to go from the refractory threshold until the maximum ethically allowed tumor volume while for tumors treated with virotherapy this was 10-32 days, and it took 13-42 days for tumors receiving both virotherapy and aPD-1. Within the first 17 days of “rescue treatment” there was a significant reduction in tumor growth for virotherapy alone (p=5*10-6) and for virotherapy plus aPD-1 (p=3.44*10-8) when compared with aPD-1 as monotherapy.

In addition, both survival and tumor growth data serve as a validation of the previously claimed aPD-1 refractory status, as the animals in the “aPD-1” group kept on receiving the antibody but experienced no additional benefit.

### Virotherapy Together With Anti-PD-1 Reshapes the Immune Microenvironment in Anti-PD-1 Refractory Tumors in Favor of Antitumor Responses

**Figure 4:**
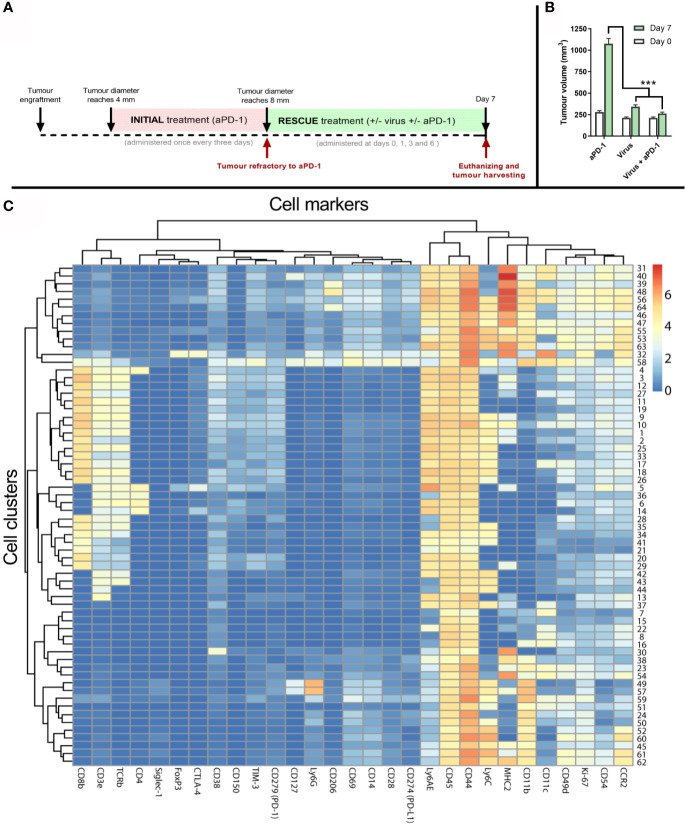
Tumor samples and analysis to study mechanism of action of the treatments. **(A)** Experimental design: 27 mice carrying B16.OVA tumors were treated with aPD-1 until they became refractory to the drug as described previously. Subsequently, those animals were assigned to groups where animals were treated with the same aPD-1 regimen (n=9), with 1x108 VP intratumorally once every three days (n=9), or both (n= 9). Four rounds of treatments were given at days 0, 1, 3 and 6 after they were considered refractory and sacrificed at day 7 for tumor collection. **(B)** Average tumor volumes (and SEM) at day 0 (when they qualified as refractory) and day 7 (when tumors were harvested). **(C)** Heatmap after the analysis of tumors by CyTOF and subsequent processing of the data by FLOWSOM providing 64 different cell clusters for immune (CD45+) cells. (Mann Whitney test; ***p < 0.001).

To generate samples to study the immune phenotype of the tumors, a similar experimental design as in **Figure 3A** was followed. However, in this separate experiment, the “rescue treatment” given after refractory status continued for seven days (**Figure 4A**). After that, tumors were collected and processed for analysis. Tumors were similar in size at baseline (day 0), when they were determined to be refractory to aPD-1. In contrast, 7 days later tumors in the “virotherapy alone” group were smaller (p<0.001). Likewise, tumors in the “virotherapy + aPD1” group were much smaller than in the “aPD1 alone” group (p<0.001), and numerically somewhat smaller than in the “virotherapy alone” group (**Figure 4B**).

To investigate if co-treatments modify the tumor microenvironment, day 7 tumors were analyzed by mass cytometry using 28 cell markers (**Supplementary Table 1**). Subsequently, 64 cell clusters were identified among all the samples by using the FlowSOM algorithm on the CD45+ fraction of the cells. Those clusters were then represented in a heatmap (**Figure 4C**) for further elucidation of corresponding cell types and phenotypes.

**Figure 5:**
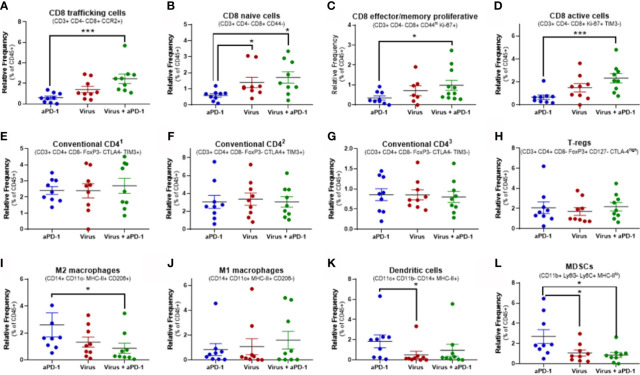
Changes in key immune populations after virotherapy assessed by mass cytometry and cluster analysis. Unbiased cell cluster generation from CD45+ fraction rendered multiple clusters that were associated to a cell type or phenotype. Relative percentage of those clusters among experimental groups were compared using Mann-Whitney test (average value and SEM included). Key markers to identify the cluster identity are indicated. **(A)** cluster 25. **(B)** cluster 41. **(C)** cluster 10. **(D)** cluster 17. **(E)** cluster 6. **(F)** cluster 14. **(G)** cluster 36. **(H)** cluster 5. **(I)** cluster 39. **(J)** cluster 58. **(K)** cluster 32. **(L)** cluster 55. *p < 0.05; ***p < 0.001.

Cell clusters resulting from mass cytometry were individually studied to determine the most likely cell type and phenotype. Those clusters with the clearest association to a specific cell population are shown in **Figure 5**.

Among all 64 cell clusters, 29 had a T-cell phenotype featuring CD45, CD3ϵ and TCRβ co-expression. 22 out of 64 were CD8+ (CD4-) while 4 were CD4+ (CD8-). Additionally, one cluster (number 4) was CD3e+ TCRb+ CD8+ CD4+ and the other two (clusters 42 and 43) were CD3e+ TCRb+ CD8- CD4-. Significant changes in relative frequencies were only observed in CD8 T-cell clusters but not in CD4, double positive or double negative T-cell clusters (**Figures 5A–H** and **Supplementary Figure 2**).

Overall, 14 different CD8 T-cell subsets were significantly increased in tumors when they received both aPD-1 and virotherapy, as compared to only 5 subsets upon virotherapy alone. Among these subsets, the combination therapy enhanced the frequency of T-cells with a phenotype linked to migration to inflamed sites (based on the CCR2 marker). In addition, these tumors harbored higher frequencies of effector/memory proliferative CD8 T cells (based on CD44 and Ki-67 markers), active and proliferating cells (based on Ki-67 and TIM-3 markers), as well as naïve T-cells (based on CD44 marker). While the combined use of aPD-1 and virotherapy consistently led to improved CD8 T-cell infiltration in the tumors, virotherapy alone did not provide the same degree of efficacy. In the same vein, the active CD8 by Treg ratio (**Figures 5D, H**) is only significantly (p= 0.032) increased when both therapies are given together.

The main focus in this experiment was to understand better in lymphoid populations in the tumor but a broad clustering of relevant myeloid populations was performed (**Figures 5I–L**).The levels of M2 macrophages and MDSCs were decreased in the tumors when virotherapy was added to the treatment. While no significant changes were observed in M1 macrophages or dendritic cells for the mentioned combination, remarkably, dendritic cells were reduced when comparing virotherapy versus aPD-1 as monotherapies.

## Discussion

Implementation of ICIs in the therapeutic armamentarium to fight cancer has changed the way in which many types of tumors are treated (2). Despite good results in a minority of patients, there is a major unmet clinical need for around 70% of the patients receiving ICIs, who are refractory to this approach (3, 7). For that reason, it is critical to improve our understanding of how ICIs work and why they fail in many patients, to tailor treatments that maximize the clinical outcome. In this regard, virotherapy has been showing promising results in terms of increasing the percentage of responders to ICIs (29, 31, 32).

Studying the interactions between ICIs and virotherapy in a preclinical model is hampered by various limitations, which probably explains the lack of work in this area, despite the obvious importance of this area. For example, currently available ICI refractory models are based on cell lines with specific mutations, but these mutations are only rarely seen in human patients (33). Ex vivo, models created out of patient-derived samples are a valuable approach to study specific causes for ICI resistance. However, it is currently not possible to have a fully operational syngeneic immune system in humanized mice models, since part of the tumor stroma – a key mediator immune responses – derives from the mouse, causing species incompatibility issues.

Our previous experiences with B16.OVA in immune competent mice (29) showed limited and heterogeneous responses to aPD-1 when used as monotherapy. Although this is somewhat surprising since both the cell line and mouse strain are clonal, also others have reported the phenomenon of variable response (30, 31). Mirroring reality in many human indications, a fraction of mice shows limited responses, others show no benefit, while a minority achieve complete responses. Inspired by clinical guidelines relating to ICIs, we proposed a model that considers tumors “refractory”, based on progression during administration of the blocking antibody. *In vivo* determination of the refractory status could have direct translatability into the clinical arena. Furthermore, a widely used model for *in vivo* Oncoimmunology studies such as the B16.OVA model for C57 mice, offers reference frameworks in terms of survival to anti-PD-1 (29, 34) and transcriptomic data (35, 36).

Anti-PD-1 refractory tumors displayed a significantly distinct transcriptional profile when compared with similar tumors naïve to aPD-1. Specifically, the gene-expression profile in these tumors represented immunosuppressive characteristics. In addition, the refractory status was validated in a different experiment, where animals considered “refractory” to the ICI showed no benefit, in terms of tumor growth control, after the antibody was administered.

In this model, the mechanism behind the refractory nature seems to support the hypothesis of resistance to therapy resulting from immune suppression in the tumor microenvironment. In particular, downregulated genes in refractory tumors correlate lack of response to aPD-1 with reduced effector T-cell function. Additionally, FOXP3, the main transcriptional factor for regulatory T cells, was upregulated in refractory tumors. Other upregulated genes in aPD-1 treated tumors might be remnants of initial effect of aPD-1, like the upregulation of genes related to complement system (37) or B-cells (38). The emergence of these genes relates to the heterogeneous process through which tumors move from immune equilibrium to escape during aPD-1 therapy.

The main purpose here was not to decipher the details that drive aPD-1 resistance, but to study if such resistance could be overcome or bypassed by the use of virotherapy. Therefore, aPD-1 refractory tumors were treated with viruses coding for TNFa and IL-2. While none of the aPD-1 refractory tumors showed signs of response to aPD-1 as monotherapy, the inclusion of virotherapy (in addition to aPD-1) triggered clear tumor growth control, and even some complete responses.

Such complete responses are remarkable, as the challenge to reject those tumors relates not only to the refractory status, but also to high and rapidly increasing tumor volume. Tumor size was around 8 times higher for the “rescue” treatment than for initial ICI. Higher tumor volume results in a narrower window for therapeutic success, because tumors are closer to the maximum ethical volume allowed.

Interestingly, rather than exploiting a different mechanism of action to drive anti-tumor responses bypassing aPD-1 resistance, virotherapy is able to tackle the suppressive status of the tumor, making them susceptible to PD-1 blockade. In this approach it is particularly relevant to take into consideration that the virotherapy used in these experiment is engineered to express TNFa and IL-2, two molecules that have shown notable ability to induce T-cell activity (22). That hypothesis explains why virotherapy together with aPD-1 renders better results than virotherapy alone, even if the tumor is not intrinsically susceptible to the antibody.

Another factor to take into account is the half-life of IgG2a antibodies in mice. The suggested time for clearing half of the antibody in adult mice is 6-8 days (39), which means that even when virotherapy is administered as monotherapy, the mice will still have some aPD-1 present in their serum. There is room to speculate that if there would be absolutely no aPD-1 in those animals treated with virotherapy only, the results in that group would be worse. The situation would be similar in human individuals refractory to aPD-1. The half-lives of pembrolizumab and nivolumab are 25 and 27 days, respectively, meaning that some antibody is present for months after the patient is determined refractory. Our results suggest that continuing inhibition of the PD-1 axis can be useful for obtaining maximum benefits of T-cell stimulating virotherapy. The combination of drugs opens the door for increased incidence and severity of adverse events. Other approaches including the combination of two immune checkpoint inhibitors like anti-PD-1 and anti-CTLA-4 are an example of that phenomenon (40). In that sense, the combination of two therapies with different mechanism of action such as an oncolytic virus and a checkpoint inhibitor has been reviewed as a safer approach (41). Another important dimension when comparing the benefits and risks of the treatments is the length of the treatment and the administration discontinuation criteria. Based on the clinically proved low toxicity of oncolytic viruses (42), the preferred approach when using them is to dose until complete response, clear tumor progression or occurrence of severe adverse events. In line with those clinical observations, animals included in this study were daily monitored by trained veterinarians and the approach was reported safe.

Focusing on the changes observed in the immune compartment after virotherapy was administered to aPD-1 refractory tumors, the main readout seems to be that the treatment increased frequencies of multiple CD8 T-cell subpopulations in the tumor, covering different developmental and functional stages. The data obtained after mass cytometry analysis of the tumor samples matches the previously proposed hypothesis on human tumors becoming refractory due to T-cell absence or dysfunction (43, 44). Virotherapy could counter the changes in CD8 cells that are making the tumors resistant to aPD-1. Upregulation of CD8 clusters with activation (CD69) and proliferation (Ki-67) markers, but low levels of exhaustion markers (TIM-3 and CTLA-4 double positives) supporting the hypothesis of an increased presence of functional antitumor adaptive responses. Many of these immune checkpoint pathways have a double nature, in the sense that they can be understood as an activation marker (as they upregulate after the T cell is activated) but also they are a source of inhibition when bound to their ligand(s), in that sense when the expression of TIM-3 and CTLA-4 was not coupled to activation and proliferation markers, it was classified of a signal of exhaustion. PD-1 expression on T cells is a relevant marker in this set-up as that molecule is the target of the checkpoint inhibitor used in this study. In that sense, cluster 3 is the subset of CD8 T cells with higher PD-1 but it is not differentially expressed in any of the testing groups. Perhaps, the initial aPD-1 treatment received by every animal to make them resistant to the antibody, normalizes the expression among all of them and that effect is not changes after subsequent treatments.

Additional understanding on the functional and phenotypical features of tumor infiltrating T cells could provide complementary insights to the impact of the therapies into this cell population. In particular, intracellular staining for different effector cytokines and transcription factors could help to determine which specific T-cell subsets are induced. Typically cytotoxic cells (sometimes called Tc1 subtype) are efficient in killing tumor and pathogen-harboring cells and they have associated markers such as intracellular IFN gamma and TNFa as well as transcription factors like EOMES, T-bet and STAT-4, while other T-cell subsets, like the Tc17 with low cytotoxic activity (45). Similarly, more in-depth characterization of T-cells would be beneficial, for example, the addition of CD45RA and CCR7 would help in terms of delineation of specific effector/memory subtypes (terminal effector memory, central memory or effector memory).

Adenovirus-induced changes in T-cell populations appear to be specific to CD8 subsets, as no significant changes were observed in conventional CD4 T cell clusters, or the regulatory CD4 T cell cluster. The fact that the active CD8 to Treg ratio is upregulated with the double treatment also points to a general environment repolarization. In addition, other cell populations suggested as candidates to mediate ICI resistance, such as MDSCs (46, 47) and M2 macrophages (7, 48) were significantly downregulated after virotherapy treatments. Regarding dendritic cells, it is remarkable to see how the presence of the viruses did not increase their presence in the tumor even if they include recognizable danger-associated molecular patterns (49). Perhaps the counterintuitive results regarding the dendritic cell compartment are related to the non-replicative nature of the virus in the model. Even if the T-cell compartment seems to be key in this model of aPD-1 resistance, it would be interesting to investigate the role of other cell types such as B cells or NK cells as well. For example, Xiao X (50) et al. identified a B cell population with high PD-1 expression and T-cell regulatory functions that could be relevant in the context of PD-1 blockade resistance.

We previously showed how the contribution of the cytokines encoded in the viruses is critical in engaging strong antitumor immune responses (23, 24). In that sense, the work described by Vredevoogd D et al (51) is specifically relevant. They showed how TNFa-related signaling could be exploited to trigger immune responses in tumors with defective responses to IFN signals. In that study, reducing the threshold for TNFa signals in the tumor caused effective immune rejection of tumors. In the present work, the presence of an immunogenic agent, such as the adenovirus, together with an increased concentration of transgenic TNFa at the tumor niche, could have a similar effect in overcoming immune resistance. When studied the impact of this virotherapeutic approach on metastases (52) and uninjected lesions (53) used in a replication competent model, it was seen how the antitumor efficacy and tumor microenvironment re-shaping also extends to those disseminated lesions. All in all, the use of this virotherapy has previously proved to be a valid approach to engage systemic antitumor responses which synergize with the systemically administered ICIs.

The work described herein focuses on studying how to increase responses of aPD-1 refractory tumors. For that purpose, a unique *in vivo* model was established and validated in the matter of resistance to the inhibitory antibody. In this model, tumors that escaped after aPD-1 administration showed a significant downregulation of immune activities mainly related with the T-cell compartment. The addition of TNFa and IL-2 armed adenoviruses to the aPD-1 regimen managed to revert the previously established resistance to aPD-1 by increasing the presence of different CD8 T cell subsets that can mediate antitumor immunity. In addition, reduced frequencies of suppressive cells (M2 macrophages and MDSCs) likely contributed to increased survival after the administration of virotherapy to aPD-1 refractory tumors. The biological mechanisms uncovered here support the continuation of ICI together with TNFa and IL-2 virotherapy, even if the tumor was originally resistant to the antibody. These preclinical results have been translated into a clinical protocol where anti-PD-(L)1 refractory tumors will be treated with TILT-123 and avelumab.

## Data Availability Statement

Complementary transcriptomic data analyzed for this study can be found in the **Supplementary Material**. Additionally, other data of this study are available from the corresponding author upon reasonable request.

## Ethics Statement

The animal study was reviewed and approved by Regional State Administrative Agency - Project Authorisation Board.

## Author Contributions

Conception and design: VC-C, AiK, and AH. Development of methodology: VC-C, DQ, JS, RH, JV, and JG-V. Acquisition of data: VC-C, DQ, IM, and JV. Analysis and interpretation of data: VC-C, DQ, JS, RH, CH, SZ, JV, JG-V, TG, and AH. Writing, review and/or revision of the manuscript: all coauthors contributed. Administrative, technical, or material support: JG-V, VB, TG, AnK, and AH. All authors contributed to the article and approved the submitted version.

## Funding

This study was supported by Marie Skłodowska-Curie Innovative Training Networks (ITN-EID VIRION H2020-MSCA-ITN-2014 project number 643130), Orion Research Foundation, Jane and Aatos Erkko Foundation, HUCH Research Funds (VTR), Sigrid Juselius Foundation, Finnish Cancer Organizations, University of Helsinki, TILT Biotherapeutics Ltd, Novo Nordisk Foundation, Päivikki and Sakari Sohlberg Foundation and the Finnish Society of Sciences And Letters. TILT Biotherapeutics Ltd was not involved in study design, collection, analysis, interpretation of data, the writing of this article or the decision to submit it for publication. We also thank Albert Ehrnrooth and Karl Fazer for research support.

## Conflict of Interest

AH is shareholder in Targovax ASA (Oslo, Norway) and in TILT Biotherapeutics Ltd. (Helsinki, Finland). AH, RH, SS, JS, and VC-C are employees of TILT Biotherapeutics Ltd. IM is employed by Orca Therapeutics, Ltd.

The remaining authors declare that the research was conducted in the absence of any commercial or financial relationships that could be construed as a potential conflict of interest.

## Publisher’s Note

All claims expressed in this article are solely those of the authors and do not necessarily represent those of their affiliated organizations, or those of the publisher, the editors and the reviewers. Any product that may be evaluated in this article, or claim that may be made by its manufacturer, is not guaranteed or endorsed by the publisher.
