# Supplementary material for: Adenovirus Armed With TNFa and IL2 Added to aPD-1 Regimen Mediates Antitumor Efficacy in Tumors Refractory to aPD-1
Source: Front Immunol. 2021 Jul 23;12:706517. doi: 10.3389/fimmu.2021.706517 (PMC8343222; doi:10.3389/fimmu.2021.706517)
Supplement: Supplementary file 1 [file DataSheet_1.pdf]

Supplementary Material

1 Supplementary Data

Supplementary Table 1. List of antibodies used for cell phenotype analysis by CyTOF

Target	Clone	Metal	Supplier	Catalog nº
CD45	30-F11	89Y	Fluidigm	3089005B
Ly6G	1A8	141Pr	Fluidigm	3141008B
CD69	H1.2F3	143Nd	Fluidigm	3143004B
CD14	Sa14-2	144Nd	Fluidigm	3144025D
CD4	RM4-5	145Nd	Fluidigm	3145002B
CD11b	M1/70	148Nd	Fluidigm	3148003B
Ly6C	HK1.4	150Nd	Fluidigm	3150010B
CD28	37.51	151Eu	Fluidigm	3151005B
CD3e	145-2C11	152Sm	Fluidigm	3152004B
CD38	90	153Eu	Biolegend	102723
CD152 (CTLA4)	UC10-4B9	154Sm	Fluidigm	3154008B
CD274 (PDL1)	10F.9G2	155Gd	Biolegend	124301
FoxP3	FJK-16s	158Gd	Fluidigm	3158003A
CD279 (PD1)	J43	159Tb	Fluidigm	3159023B
CD206	C068C2	160Gd	Biolegend	141701
CD366 (TIM3)	RMT3-23	162Dy	Fluidigm	3162029B
CD54	YN1/1.7.4	163Dy	Fluidigm	3163020B
Ly6A/E	D7	164Dy	Fluidigm	3164005B
CD192 (CCR2)	475301	165Ho	RnD systems	MAB55381-SP
CD150	TC15-12F12.2	167Er	Fluidigm	3167004B
CD8b	53-6.7	168Er	Biolegend	100701
TCRb	H57-597	169Tm	Fluidigm	3169002B
CD44	IM7	171Yb	Fluidigm	3171003B
ki-67	B56	172Yb	Fluidigm	3172024B
MhC-2	M5/114.15.2	174Yb	Fluidigm	3174003B
CD127	A7R34	175Lu	Fluidigm	3175006B
CD49d (a4-integrin)	R1-2	176Yb	Biolegend	103610
CD11c	N418	209Bi	Fluidigm	3209005B

Supplementary Table 2. Significantly regulated genes in treatment naïve vs aPD-1 treated progressing tumors. (Differences in gene regulations were taken into account if fold change was ≤-2 or ≥2, with a q-value ≤ 0.001).

List of upregulated genes

GeneID	Expression Ratio (aPD-1 vs Mock)	q-value	Symbol
100043387	429.008	1.96E-52	Gm14305
666184	109.449	1.21E-16	Gm15080
20927	98.387	3.49E-15	Abcc8
11808	55.841	2.74E-09	Apoa4
403395	49.194	2.56E-08	Clec3a
93732	37.228	6.92E-12	Acox2
56636	29.535	5.88E-60	Fgf21
100503613	29.250	2.43E-05	5031434C07Rik
667666	28.665	2.99E-05	Zfp600
16691	25.210	5.68E-93	Krt8
239790	21.938	3.53E-07	Ostn
100322896	21.273	5.69E-07	Dthd1
100040880	20.994	4.59E-04	Gm3020
545648	20.728	5.05E-04	Gm13272
545136	20.608	9.16E-07	Fam186b
209601	18.614	5.13E-16	Erich3
386463	12.423	7.14E-98	Cdsn
100039672	11.666	1.12E-05	Msmp
245468	10.214	3.74E-20	Pnma3
11997	9.783	1.25E-30	Akr1b7
385377	8.978	1.09E-80	Pnma5
18054	8.393	3.03E-15	Ngp
241118	8.144	6.50E-08	Asic4
69169	7.692	5.12E-12	Fcmr
101613	6.980	6.83E-04	Nlrp6
545007	6.600	4.23E-04	Gm5796
317677	6.519	1.12E-04	C1s2
67937	6.346	7.19E-14	Tmem59l
20495	5.651	9.61E-11	Slc12a1
12482	5.267	3.18E-12	Ms4a1
20616	5.170	1.37E-08	Snap91
12839	4.832	2.42E-39	Col9a1

GeneID	Expression Ratio (aPD-1 vs Mock)	q-value	Symbol
545650	4.617	1.10E-04	Gm13277
18419	4.539	3.52E-04	Otog
70771	4.487	8.84E-04	Gpr173
60345	4.055	2.25E-22	Nrip2
12478	3.989	2.34E-08	Cd19
194974	3.797	5.44E-05	Sun3
320581	3.773	9.37E-04	Idi2
81879	3.719	6.82E-22	Tfcp2l1
243369	3.582	1.28E-08	Sspo
237625	3.472	4.17E-36	Pla2g3
78709	3.469	1.50E-13	Spink8
68625	3.461	2.46E-12	Cfap57
246788	3.419	7.68E-04	Trpv3
17068	3.276	5.54E-06	Ly6d
72393	3.263	1.65E-07	Faim2
225583	3.257	1.38E-04	A730017C20Rik
17183	3.244	3.25E-10	Matn4
330122	3.143	6.66E-07	Cxcl3
58217	3.122	3.36E-05	Trem1
433804	3.096	1.32E-28	Gm13154
58860	3.028	8.48E-04	Adamdec1
245610	2.984	1.89E-60	Nxf3
11567	2.881	1.52E-06	Avil
15101	2.865	1.94E-07	H60a
237868	2.864	1.77E-04	Sarm1
100417831	2.848	1.78E-14	Gm18853
12902	2.750	3.34E-06	Cr2
100102	2.749	5.32E-13	Pcsk9
26570	2.737	9.01E-71	Slc7a11
12931	2.729	2.21E-86	Crlf1
23966	2.718	2.67E-31	Tenm4
16336	2.711	1.75E-04	Insl3

GeneID	Expression Ratio (aPD-1 vs Mock)	q-value	Symbol
76142	2.707	2.08E-06	Ppp1r14c
330627	2.659	1.68E-08	Trim66
11941	2.620	1.24E-04	Atp2b2
100039257	2.589	5.04E-139	Tmem254b
100504375	2.546	2.75E-13	Gm15880
18985	2.472	1.02E-06	Pou2af1
59083	2.438	3.39E-05	Fetub
76886	2.396	1.74E-05	Fam81a
70370	2.357	7.69E-08	Fbln7
14990	2.353	8.89E-47	H2-M2
11549	2.327	2.01E-04	Adra1a
13982	2.322	1.69E-58	Esr1
69994	2.316	1.36E-17	Rsc1a1
320088	2.292	4.91E-04	C030034L19Rik
70045	2.283	6.85E-14	2610528A11Rik
24113	2.271	1.06E-05	Vax2
394435	2.239	2.31E-05	Ugt1a6b
15051	2.226	8.26E-05	H2-T9
228775	2.204	1.35E-123	Trib3
106407	2.173	1.77E-10	Slc51a
93715	2.136	8.38E-04	Pcdhga7
110312	2.131	4.52E-04	Pmch
12950	2.120	1.43E-163	Hapln1
76960	2.117	3.08E-54	Bcas1
72685	2.099	2.87E-05	Dnajc6
15417	2.063	1.31E-06	Hoxb9
228993	2.051	1.11E-11	Slc17a9
17394	2.047	5.90E-15	Mmp8
12903	2.041	2.13E-05	Crabp1
216188	2.036	6.94E-214	Aldh1l2
20371	2.029	8.10E-17	Foxp3
15426	2.014	8.37E-04	Hoxc8

List of downregulated genes

GeneID	Expression Ratio (aPD-1 vs Mock)	q-value	Symbol
241431	2.26E-04	1.61E-212	Xirp2
17884	3.55E-04	0.00E+00	Myh4
17882	0.001	1.49E-95	Myh2
76757	0.001	6.06E-78	Trdn
17879	0.001	0.00E+00	Myh1
21957	0.002	0.00E+00	Tnnt3
69585	0.002	2.04E-42	Hfe2
17901	0.002	0.00E+00	Myl1
11459	0.002	0.00E+00	Acta1
58916	0.002	5.51E-70	Myot
233199	0.003	1.86E-300	Mybpc2
109272	0.004	7.88E-141	Mybpc1
53311	0.004	6.47E-23	Mybph
19293	0.004	1.07E-299	Pvalb
17930	0.006	8.29E-93	Myom2
233246	0.006	3.55E-31	Ano5
53318	0.007	3.63E-85	Pdlim3
140491	0.007	1.03E-27	Ppp1r3a
223513	0.007	2.00E-14	Abra
58522	0.007	6.31E-14	Trim54
16514	0.007	1.12E-13	Kcnj11
21925	0.008	0.00E+00	Tnnc2
100039116	0.008	3.45E-13	Mup14
66106	0.008	3.71E-24	Smpx
666794	0.009	3.83E-12	Rbm24
21393	0.010	2.77E-100	Tcap
17878	0.010	7.74E-11	Myf6
140781	0.010	1.70E-10	Myh7
244923	0.010	1.04E-19	Klhl31
11937	0.011	0.00E+00	Atp2a1
17306	0.011	7.82E-52	Sypl2
17189	0.012	8.81E-65	Mb
11474	0.013	0.00E+00	Actn3
17885	0.013	2.49E-08	Myh8
12391	0.015	1.48E-07	Cav3
320502	0.017	8.62E-13	Lmod3
22437	0.017	3.20E-69	Xirp1
17927	0.017	1.04E-06	Myod1
17928	0.017	1.04E-06	Myog
100041449	0.018	2.01E-06	Cyp3a59
13009	0.019	8.08E-22	Csrp3
99738	0.020	4.72E-11	Kcnc4
381485	0.020	3.94E-06	Trim55
215031	0.020	3.94E-06	Vgll2
100039028	0.021	7.86E-06	Mup11
68515	0.022	1.51E-05	Myadml2
21953	0.024	7.76E-257	Tnni2
319168	0.024	3.00E-05	Hist1h2ah
13628	0.025	2.47E-102	Eef1a2
16495	0.027	1.46E-15	Kcna7
76294	0.027	3.57E-22	Asb5
100504518	0.027	2.83E-44	3425401B19Rik
68460	0.030	1.83E-07	Dhrs7c
230883	0.030	2.36E-04	Aadacl3
16814	0.030	2.36E-04	Lbx1
24131	0.031	2.39E-131	Ldb3
100040972	0.033	4.74E-04	Tceal7
234072	0.033	4.74E-04	Adprhl1
545798	0.033	4.74E-04	Tmem233
27281	0.037	9.63E-04	Hrasls
399548	0.038	1.35E-30	Scn4b
229665	0.039	7.22E-83	Ampd1
57339	0.044	6.72E-26	Jph1
78977	0.044	2.55E-05	Popdc3
75581	0.047	5.24E-05	Yipf7
18175	0.049	3.73E-138	Nrap
17926	0.049	4.69E-23	Myoc
17907	0.052	0.00E+00	Mylpf
381339	0.055	1.59E-07	Tmem182
68802	0.056	2.55E-32	Mypn
24117	0.058	3.26E-07	Wif1
93677	0.059	1.12E-24	Lmod2
268859	0.060	3.37E-21	Rbfox1
16716	0.060	1.32E-12	Ky
68701	0.060	9.47E-10	Ppp1r27
17897	0.060	4.50E-04	Myl3
76722	0.061	2.42E-32	Ckmt2
22138	0.063	0.00E+00	Ttn
20771	0.066	9.25E-04	Mucl1
68016	0.076	3.87E-16	Murc
434246	0.076	1.59E-35	Trim72
19716	0.077	2.03E-05	Bex1
110880	0.080	1.62E-31	Scn4a
12299	0.082	8.38E-17	Cacng1
101359	0.083	4.90E-05	Prrt4
21380	0.089	9.98E-05	Tbx1
225638	0.091	2.03E-11	Alpk2

GeneID	Expression Ratio (aPD-1 vs Mock)	q-value	Symbol
12715	0.092	0.00E+00	
76469	0.093	6.71E-171	
268807	0.098	2.73E-07	
50874	0.099	2.76E-16	
72330	0.108	9.21E-27	
116904	0.109	1.87E-26	
93897	0.111	8.23E-04	
12733	0.111	8.23E-04	
26549	0.113	3.01E-11	
98660	0.116	4.83E-83	
228003	0.117	1.14E-80	
14199	0.117	6.27E-167	
107765	0.119	9.24E-46	
105349		6.31E-14	
142687	0.121	8.91E-06	
70100	0.124	1.28E-05	
74376	0.125	4.19E-34	
319167	0.131	2.42E-04	
69700	0.133	3.68E-07	
59006	0.133	3.44E-05	
16979	0.133	3.34E-04	
72585	0.136	4.61E-05	
54612	0.138	7.26E-07	
59011	0.141	2.85E-43	
11472	0.142	3.15E-74	
241113	0.153	9.69E-06	
545649	0.153	4.43E-11	
67252	0.154	5.71E-29	
11722	0.156	1.70E-10	
11464	0.158	5.69E-15	
240892	0.166	1.60E-07	
244757	0.166	1.97E-05	
12180	0.167	5.41E-27	
53412	0.175	1.14E-16	
17996	0.177	6.03E-226	
434866	0.180	7.03E-07	
11870	0.183	3.64E-25	
12350	0.184	3.44E-258	
27206	0.185	3.53E-04	
72713	0.186	2.70E-05	
22004	0.186	7.63E-297	
16002	0.186	6.78E-15	
19699	0.192	1.64E-09	
269862	0.196	6.67E-04	
242653	0.196	6.67E-04	
18670	0.204	6.05E-11	
29818	0.206	1.05E-83	
27273	0.209	5.12E-35	
57435	0.211	5.64E-29	
13346	0.211	0.00E+00	
57262	0.214	0.00E+00	
17906	0.214	9.21E-06	
77673	0.214	9.21E-06	
12643	0.215	3.61E-11	
100041874	0.216	7.36E-05	
231148	0.222	6.38E-09	
66959	0.222	6.38E-09	
100043335	0.222	8.45E-04	
15464	0.223	1.04E-19	
319151	0.224	2.54E-04	
63993	0.229	1.08E-09	
64103	0.230	7.86E-09	
73712	0.230	1.40E-11	
231470	0.231	2.15E-04	
100504346	0.232	9.06E-06	
100504239	0.232	9.06E-06	
17929	0.233	5.49E-53	
238564	0.237	2.24E-34	
240322	0.237	5.43E-05	
14944	0.242	3.20E-07	
53857	0.245	1.08E-14	
237611	0.247	8.69E-07	
17528	0.252	6.45E-79	
235379	0.253	6.86E-04	
68760	0.256	1.56E-17	
70887	0.260	4.53E-04	
100048884	0.271	2.11E-24	
100039192	0.272	1.12E-163	
12322	0.273	7.32E-21	
50795	0.279	2.71E-16	
574437	0.287	6.78E-12	
66175	0.287	7.81E-26	
17153	0.288	4.34E-07	
20190	0.289	2.70E-107	
242702	0.295	4.15E-05	
330166	0.297	2.70E-05	
78785	0.309	1.97E-14	

GeneID	Expression Ratio (aPD-1 vs Mock)	q-value	Symbol
12862	3.15E-01	2.04E-31	Cox6a2
68854	3.15E-01	4.20E-07	Asb11
98363	0.315	4.20E-07	Efhd1
72607	0.316	2.75E-11	Usp13
574405	0.316	1.41E-04	DXBay18
77018	0.323	1.28E-04	Col25a1
18829	0.325	4.98E-10	Ccl21a
21401	0.328	2.43E-08	Tcea3
74954	0.331	2.75E-05	4930503E14Rik
78321	0.332	3.90E-117	Ankrd23
64082	0.332	7.62E-04	Popdc2
320111	0.334	3.22E-41	Prr18
22354	0.336	1.56E-47	Vipr1
228785	0.337	4.65E-32	Mylk2
330483	0.339	3.89E-06	Ceacam16
74574	0.348	6.57E-05	Lvrn
56012	0.348	5.51E-34	Pgam2
170947	0.349	7.47E-05	Myoz3
74165	0.350	4.16E-05	Fbxl22
20167	0.354	3.65E-20	Rtn2
100134861	0.356	6.62E-16	ChkbCpt1b
14582	0.363	1.39E-07	Gfi1b
320563	0.364	1.54E-09	Islr2
16353	0.367	2.60E-04	Ipw
100503386	0.369	2.73E-06	Tpbgl
93710	0.372	5.65E-07	Pcdhga2
13717	0.374	7.54E-90	Eln
231238	0.375	2.02E-12	Sel1l3
12372	0.379	1.13E-57	Casq1
72948	0.388	8.30E-05	Tppp
626009	0.391	4.87E-06	Gm6644
217517	0.391	7.94E-10	Stxbp6
19126	0.391	9.85E-04	Prom1
114332	0.392	2.39E-93	Lyve1
20379	0.392	6.16E-47	Sfrp4
11537	0.402	6.42E-22	Cfd
240873	0.410	5.13E-09	Tnfsf18
11689	0.415	1.80E-16	Alox5
76477	0.415	4.68E-07	Pcolce2
12424	0.417	0.00E+00	Cck
18553	0.421	1.46E-15	Pcsk6
118449	0.422	1.65E-22	Synpo2
68794	0.425	2.19E-43	Flnc
13497	0.425	8.08E-07	Drp2
67971	0.430	3.01E-34	Tppp3
11450	0.431	8.30E-06	Adipoq
18682	0.432	1.57E-06	Phkg1
73942	0.436	1.75E-05	Fam151b
677289	0.438	1.63E-06	Prr33
11733	0.443	7.13E-10	Ank1
16642	0.444	2.26E-04	Klrc2
81877	0.444	3.50E-67	Tnxb
16012	0.446	3.68E-25	Igfbp6
56747	0.448	0.00E+00	Sez6l
654432	0.451	2.47E-44	Gm7334
17221	0.452	4.65E-04	Cd46
56429	0.454	2.25E-77	Dpt
53973	0.456	4.70E-07	Cyp3a41a
71912	0.457	1.05E-06	Jsrp1
11668	0.458	4.30E-08	Aldh1a1
21930	0.467	9.97E-09	Tnfaip6
57429	0.467	1.15E-20	Sult5a1
30937	0.468	4.44E-12	Lmcd1
100039246	0.471	8.56E-06	Plac9b
236749	0.471	3.95E-04	Gm4907
68458	0.471	4.41E-04	Ppp1r14a
14955	0.471	3.05E-41	H19
100040591	0.473	0.00E+00	Kcnj13
216616	0.473	1.52E-42	Efemp1
110257	0.475	0.00E+00	Hba-a2
11811	0.475	1.96E-09	Apobec2
67888	0.475	1.22E-07	Tmem100
56216	0.476	8.50E-04	Stx1b
14734	0.476	4.96E-22	Gpc3
21955	0.478	8.34E-05	Tnnt1
100503605	0.481	0.00E+00	Hbb-bs
21922	0.484	7.94E-14	Clec3b
16011	0.485	6.05E-116	Igfbp5
16502	0.485	7.21E-04	Kcnc1
11435	0.486	4.47E-05	Chrna1
100039008	0.486	7.06E-12	Mup10
13587	0.486	1.85E-17	Ear2
15122	0.487	0.00E+00	Hba-a1
105387	0.490	6.61E-08	Akr1c14
100689	0.491	1.19E-06	Spon2
14943	0.493	1.04E-15	Gzmf
56642	0.500	2.91E-09	Ankrd2

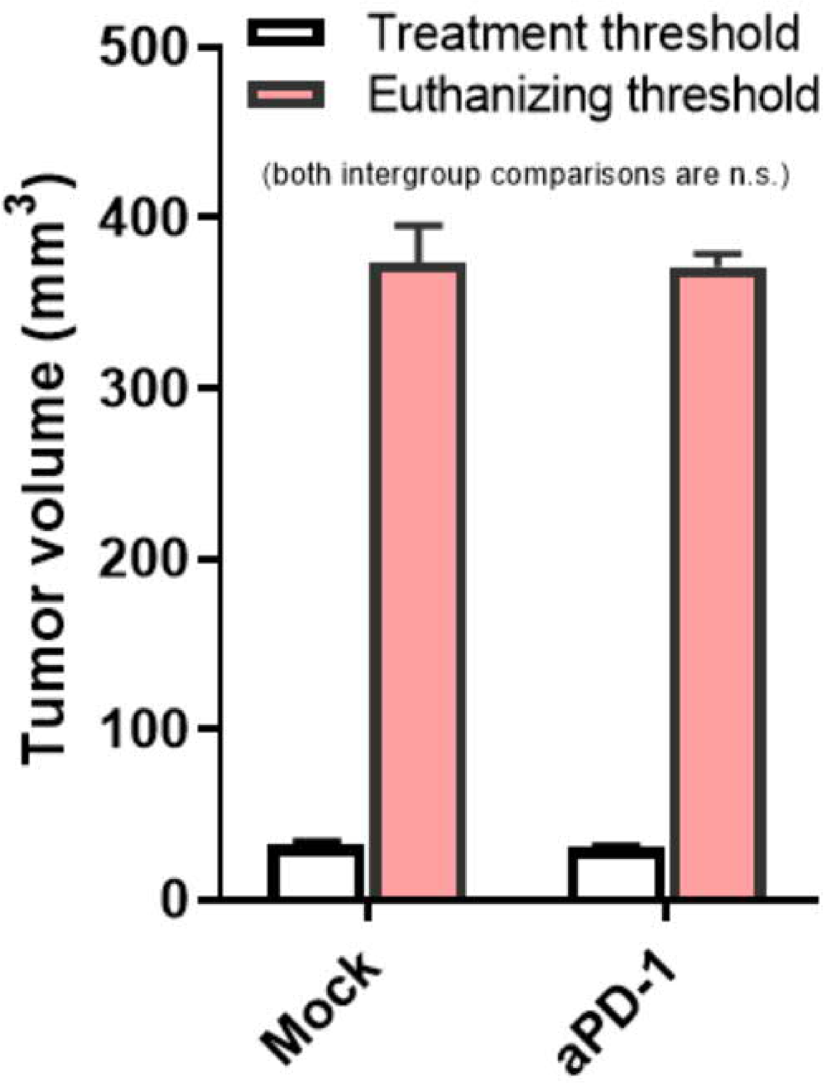

Supplementary figure 1. Average tumor volume (with SEM) at treatment threshold and euthanizing threshold. (Mann Whitney test; n.s. $p>0.05$)

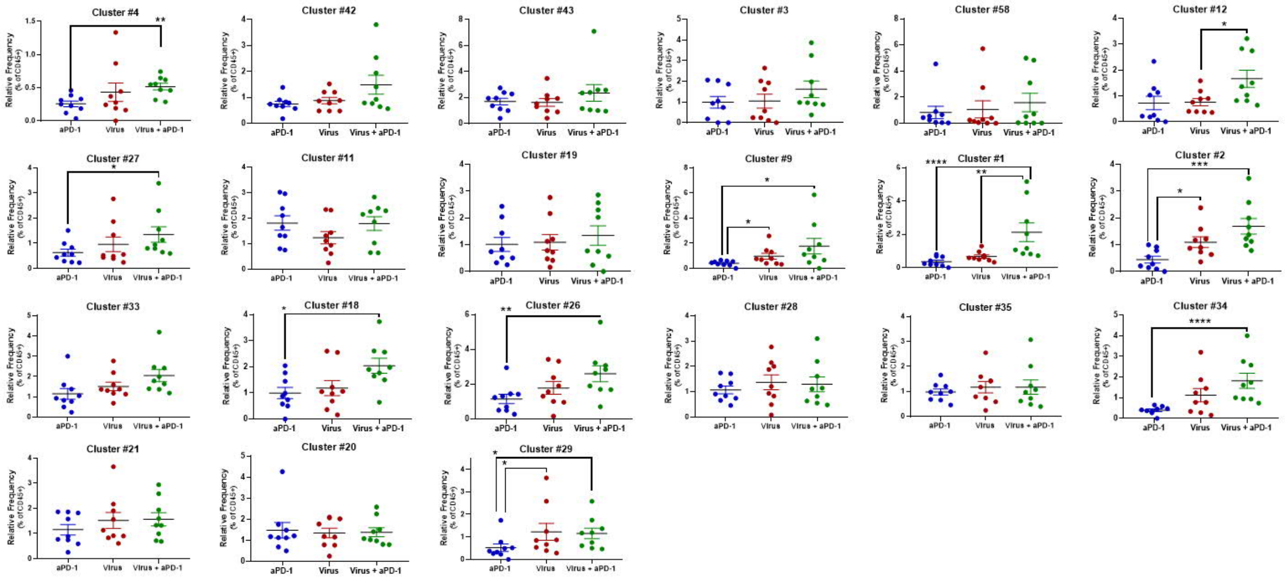

Supplementary figure 2. Additional T-cell cluster comparisons from treated aPD-1 refractory tumors. (Representations include average and SEM. Mann-Whitney test was used to assess significances. *p<0.05, **p<0.01 and ***p<0.001, ****p<0.0001).
